# Healthcare worker experiences with Option B+ for prevention of mother-to-child HIV transmission in eSwatini: findings from a two-year follow-up study

**DOI:** 10.1186/s12913-019-3997-1

**Published:** 2019-04-02

**Authors:** Abby L. DiCarlo, Averie Baird Gachuhi, Simangele Mthethwa-Hleta, Siphesihle Shongwe, Thabo Hlophe, Zachary J. Peters, Allison Zerbe, Landon Myer, Nontokozo Langwenya, Velephi Okello, Ruben Sahabo, Harriet Nuwagaba-Biribonwoha, Elaine J. Abrams

**Affiliations:** 1 ICAP at Columbia University, Mailman School of Public Health, New York, NY USA; 2 Ministry of Health, Mbabane, eSwatini; 3 Division of Epidemiology & Biostatistics, School of Public Health & Family Medicine, University of Cape Town, Cape Town, South Africa; 4 Centre for Infectious Diseases Epidemiology & Research, School of Public Health & Family Medicine, University of Cape Town, Cape Town, South Africa; 5 Vagelos College of Physicians & Surgeons, Columbia University, New York, NY USA

**Keywords:** HIV, Option B +, Prevention of mother-to-child transmission, Health care workers, Universal antiretroviral therapy

## Abstract

**Background:**

Prevention of mother-to-child transmission (PMTCT) across sub-Saharan Africa has rapidly shifted towards Option B+, an approach in which all HIV+ pregnant and breastfeeding women initiate lifelong antiretroviral therapy (ART) independent of CD4+ count. Healthcare workers (HCW) are critical to the success of Option B+, yet little is known regarding HCW acceptability of Option B+, particularly over time.

**Methods:**

Ten health facilities in the Manzini and Lubombo regions of eSwatini transitioned from Option A to Option B+ between 2013 and 2014 as part of the Safe Generations study examining PMTCT retention. Fifty HCWs (5 per facility) completed questionnaires assessing feasibility and acceptability: (1) prior to transitioning to Option B+, (2) two months post transition, and (3) approximately 2 years post Option B+ transition. This analysis describes HCW perceptions and experiences two years after transitioning to Option B+.

**Results:**

Two years after transition, 80% of HCWs surveyed reported that Option B+ was easy for HCWs, noting that it was particularly easy to explain and coordinate. Immediate ART initiation also reduced delays by eliminating need for laboratory tests prior to ART initiation. Additionally, HCWs reported ease of patient follow-up (58%), documentation (56%), and counseling (58%) under Option B+. Findings also indicate that a majority of HCWs reported that their workloads increased under Option B+. Sixty-eight percent of HCWs at two years post-transition reported more work under Option B+, specifically noting increased involvement in adherence counseling, prescribing/monitoring medications, and appointment scheduling/tracking. Some HCWs attributed their higher workloads to increased client loads, now that all HIV-positive women were initiated on ART. New barriers to patient uptake, and issues related to retention, adherence, and follow-up were also noted as challenges face by HCW when implementing Option B+.

**Conclusions:**

Overall, HCWs found Option B+ to be acceptable and feasible while providing critical insights into the practical issues of universal ART. Further strengthening of the healthcare system may be necessary to alleviate worker burden and to ensure effective monitoring of client retention and adherence. HCW perceptions and experiences with Option B+ should be considered more broadly as countries implement Option B+ and consider universal treatment for all HIV+ individuals.

**Trial registration:**

http://clinicaltrials.gov NCT01891799, registered on July 3, 2013.

**Electronic supplementary material:**

The online version of this article (10.1186/s12913-019-3997-1) contains supplementary material, which is available to authorized users.

## Background

Since 2013, prevention of mother-to-child transmission (PMTCT) across sub-Saharan Africa has rapidly shifted towards Option B+, an approach in which all HIV-positive pregnant and breastfeeding women initiate lifelong antiretroviral treatment (ART) independent of CD4+ count. Ninety-five percent of Global Plan priority countries have endorsed Option B+, and the percentage of HIV-positive pregnant women receiving ART has increased drastically from 36% in 2009 to 77% in 2015 [1]. Between 2009 and 2015, the number of new pediatric HIV infections in the 21 Global Plan priority countries dropped by 60%, with an estimated 110,000 new pediatric infections in 2015 [1]. To date, universal ART during pregnancy appears to be a promising and effective PMTCT strategy. However, the success of Option B+ is dependent upon its long-term sustainability, and new concerns have arisen regarding adherence and retention in care under Option B+, particularly after delivery [2–8].

Maternal, newborn, and child health (MNCH) services are considered the backbone of the health system in sub-Saharan Africa [9]. The evolution of PMTCT has dramatically changed the nature of MNCH services over time, and World Health Organization (WHO) PMTCT guidance has advanced quickly. Early WHO recommendations on antiretroviral medications (ARVs) for pregnant women have evolved from the provision of single-dose nevirapine at delivery to short-course zidovudine (AZT) during pregnancy, and then to Options A and B, which relied on laboratory and clinical screening to determine the appropriate ARV regimen for each pregnant and breastfeeding woman, to Option B+, which provides universal ART to all who test HIV-positive [10, 11]. While past PMTCT approaches required some level of integration into MNCH services in a time-limited capacity, the successful implementation Option B+ necessitates a more complete integration of PMTCT, HIV, and MNCH services and has shifted the paradigm of PMTCT from a time-limited intervention during pregnancy and delivery to an ongoing practice of care. In these ways, Option B+ requires a new level of service delivery beyond traditional MNCH services [12].

The introduction of Option B+ and rapid scale-up of PMTCT services in sub-Saharan Africa has occurred within a health system with well-documented health workforce shortages, rapidly changing national health policies, and other chronic issues [13–16]. The global uptake of Option B+ has increased patient loads and contributed to increased burdens on healthcare workers (HCWs). Strategies such as task-shifting, increasing the responsibilities of nursing staff, and the expansion of community and lay health workers have been implemented to address inadequacies in the health system infrastructure, but there has been relatively little research on the Option B+ scale-up from the perspectives of those implementing and delivering care [17]. HCWs are on the frontlines of health initiatives and are uniquely positioned to directly influence patient engagement and retention in care, and to provide practical feedback on health interventions [18]. However, few studies have explored the acceptability of Option B+ among healthcare workers [14, 19], particularly over time. HCW perspectives and experiences are integral to health service delivery, and will be especially important as global programs move to implementing universal treatment for all individuals living with HIV infection [20].

The Kingdom of eSwatini is a small country of about 1.25 million people and is at the epicenter of the global HIV/AIDS pandemic, with the highest HIV prevalence globally (28.8% of individuals ages 15–49) [21]. Additionally, eSwatini is classified as a Human Resources for Health “crisis country” by the World Health Organization, with a staff-population ratio of 1.69 to 1000, well below the WHO minimum recommendation of 2.5 staff (doctors, nurses, midwives) per 1000 people [22]. In 2013, the Ministry of Health (MOH) in the Kingdom of eSwatini partnered with ICAP in a USAID-funded implementation science research study, called Safe Generations, to evaluate the impact of Option B+ on retention outcomes at ten health facilities in eSwatini [23]. This study also assessed how HCWs managed and experienced implementation of the Option B+ strategy. This analysis describes the perceptions and experiences of HCW at two months and at two years after transitioning to Option B+.

## Methods

### The Safe Generations study

From September 2013 through June 2014, ten health facilities across the Manzini and Lubombo regions in eSwatini transitioned from Option A to Option B+, using a stepped-wedge design [23]. Under Option A, HIV-positive pregnant women received ARV prophylaxis or ART as determined by their immunologic and clinical status; under Option B+, all HIV-positive pregnant women initiated lifelong ART at the first antenatal visit, i.e., at diagnosis for newly-diagnosed HIV-positive women or at entry into PMTCT care for women already diagnosed as HIV-positive. At the start of the study all facilities offered Option A as standard of care and one facility transitioned to offer Option B+ every four weeks. Study staff assembled mentoring teams, consisting of one nurse and one adherence/psychosocial (APS) counselor, to provide facility-based HCWs with intensive mentoring and training on Option B+. Prior to transitioning to Option B+, HCWs at each facility were trained on standard operating procedures, including counseling, medication, adherence, record-keeping, disclosure, delivery, breastfeeding, and infant testing. Study staff also provided HCWs with flip charts for use with patients, in order to guide counseling and education on Option B+. Study mentoring teams remained on site for four weeks following the transition to Option B+ in order to provide implementation support and guidance. Study mentoring teams continued to provide any necessary support for the duration of the study and teams received monthly retraining to ensure their implementation support remained consistent over time.

### Study design: healthcare worker acceptability study

This study explored how healthcare workers at the Safe Generations study facilities experienced the implementation of the Option B+ strategy across three time points: 1) before transition to Option B+, (2) two months post-transition, and (3) approximately two years post-transition. Findings from data collected at the first time point (before transitioning to Option B+) have been previously presented and are not included in this analysis [24].

Study staff recruited fifty HCWs who were actively involved in providing PMTCT services, primarily nurses, nurse midwives, and mentor mothers/peer counselors, to participate in the study. Five HCWs were recruited from each of the ten study facilities. Eligibility criteria included being at least 18 years old, able to speak English, willing and able to provide signed informed consent, willing to adhere to study procedures and follow-up interviews, and actively delivering PMTCT services at a participating study site. The study was introduced to the HCWs by their local supervisor (sister-in-charge); those interested in participating then met with the study research assistant to review study procedures. All eligible participants completed a written informed consent and were assured that their decision around participation in the study would not impact their employment status. Participants were expected to complete survey questionnaires at three time points: (1) before transition to Option B+, (2) two months post-transition, and (3) approximately two years post-transition. Every effort was made to include the same HCWs in each survey time point; however, in the event that was not possible, replacement HCWs were recruited, as described above, for subsequent rounds using the same eligibility criteria.

The study protocol, data collection instruments, and consent forms were approved by the Columbia University Medical Center Institutional Review Board and the Scientific and Ethics Committee at the eSwatini Ministry of Health.

### Data collection

Participants completed survey questionnaires assessing feasibility and acceptability at three time points across transition from Option A to Option B+. Surveys were designed to capture how HCWs characterized the acceptability, feasibility, and advantages and challenges of Option B+, and included questions related to perceived barriers to PMTCT implementation, attitudes towards Option B+, and perceptions of PMTCT client uptake (Additional files 1 and 2). Developed in close collaboration with the eSwatini study team, the survey instrument was then piloted and revised through an iterative process with local staff and HCWs. Surveys at all time points covered the same topics; several minor additions were made to the two-year survey in order to further explore salient themes from the first round of findings. Additions to the two-year survey were kept to an absolute minimum and were discussed and refined by three research team members. As a result, the two-year survey directly assesses the general ease of Option B+ for HCWs, and the ease of follow-up and documentation under Option B+; previous iterations of the survey did not directly measure these factors. Surveys included a combination of close-ended (dichotomous choice, multiple choice, and scaled) and open-ended questions. Open-ended questions included opportunities to expand on close-ended questions (“please explain”), as well as stand-alone questions designed to elicit more descriptive and complex responses.

Interviews took place in a private space within the facility to ensure confidentiality and privacy. Questionnaires were administered by research assistants and responses to questions were recorded on paper-based files by the research assistant conducting the interview. Participant confidentiality was maintained by the use of unique study ID numbers throughout the study.

### Data analysis

Frequency statistics for closed-ended questions were produced in SAS 9.3. As some closed-ended questions were designed to include multiple responses, percentages for close-ended questions do not always add up to 100. To assess significant statistical differences in nominal variables between time points, we used McNemar’s Exact test.

Coding software (ATLAS.ti Version 1.0.38) was used for qualitative analysis. Open-ended questions were coded using content analysis [25] to identify themes and patterns, with preliminary codes developed along significant lines of inquiry. Questionnaires were read and re-read to develop a coding framework, which was discussed, refined, and verified for consensus among the research team at each time point. Open-ended question analysis was an ongoing and iterative process at each time point, which allowed for the possible emergence of new themes over time.

Qualitative and quantitative data were compared and analyzed together to identify salient themes across both types of data.

## Results

Fifty HCWs were enrolled across the ten health facilities (5 per facility). The majority (70%) of the sample was maintained over two years, with 15 HCWs being replaced at two years post-transition. The replacement of participants at two years post-transition did not result in significant differences in the distribution of professional roles and demographics at two months versus two years post-transition (Table 1). Participants in the two-month post-transition survey included nurses/nurse midwives (52%), mentor mothers/peer counselors (26%), and expert clients (12%). Participants in the two-year post-transition survey included nurses/nurse midwives (54%), mentor mothers/peer counselors (32%), and expert clients (10%). The median age and gender distribution remained largely the same across the two samples.

**Table 1:** Comparison of healthcare worker samples at two months and two years post-implementation of Option B+

	2 months post-transition (*N* = 50)	2 years post-transition (*N* = 50)^a^
Age (range)	36 (31–42)	36 (31–43)
Female	46 (92%)	47 (94%)
Male	4 (8%)	3 (6%)
Professional roles		
Nurse midwives	19 (38%)	22 (44%)
Nurses	7 (14%)	5 (10%)
Mentor mothers	7 (14%)	9 (18%)
Peer counselors	6 (12%)	7 (14%)
Expert clients	6 (12%)	5 (10%)
Other (adherence officers, nursing assistants, phlebotomist)	5 (10%)	2 (4%)

^a^Two-year sample includes 35 original participants and 15 replacement participants

Overall findings demonstrate that HCWs found Option B+ to be highly acceptable and preferable to Option A. Two years after transition, 80% reported that Option B+ was easy for HCWs (Table 2), noting that it was particularly easy to explain and coordinate. Immediate ART initiation also reduced delays by eliminating the need for laboratory tests prior to ART initiation. Additionally, HCWs reported ease of patient follow-up (58%), documentation (56%), and counseling (58%) under Option B+.

**Table 2:** Healthcare worker responses to assessment of Option B+

	2 months post-transition (*N* = 50)	2 years post-transition (*N* = 50)
Overall, do you think Option B+ is easy or hard for HCWs?		
Easy	n/a^a^	40 (80%)
Hard		10 (20%)
Do you think following up with patients who initiated ART under Option B+ is easy or hard for healthcare workers?		
Easy	n/a^a^	29 (58%)
Hard		21 (42%)
Do you think documentation of patient care under Option B+ is easy or hard for healthcare workers?		
Easy	n/a^a^	28 (56%)
Hard		21 (42%)
No Answer		1 (2%)
In your opinion is it easy or hard to counsel patients about Option B +?		
Easy	25 (50%)	29 (58%)
Hard	24 (48%)	21 (42%)
No Answer	1 (2%)	
Do you think you have more work or less work now (since implementation of Option B+)?		
More Work	31 (62%)	34 (68%)
Less Work	9 (18%)	8 (16%)
Same Amount	8 (16%)	8 (16%)
Do you find that women commonly refuse to start ART the same day they are diagnosed with HIV?		
Yes	16 (32%)	7 (14%)
No	33 (66%)	43 (86%)
No Answer	1 (2%)	

^a^Question was an addition to two year survey and not directly assessed at two months post-transition

nb

Qualitative themes that were identified across both post-transition time points included (1) the advantages of Option B+, including: the elimination of ART eligibility requirements, ease of explaining Option B+ to patients, simple and effective counseling, and decreased institutional barriers to care; and (2) the challenges of Option B+, including: increased worker burden, new barriers to acceptability for patients, and challenges related to retention, adherence, and follow-up.

### Option B+ advantages

#### Elimination of eligibility requirements

Immediate ART initiation for all HIV-positive pregnant women, regardless of CD4+ count, was frequently cited as a primary logistical advantage of Option B+ at both two months and two years post-transition. The elimination of previously required CD4+ eligibility requirements for ART initiation simplified the PMTCT process for HCWs and their patients. Under Option B+, HCWs were no longer required to send off blood samples for laboratory studies including CD4+ cell count before initiating patients on ART, and therefore no longer needed to manage supplies or staff members related to laboratory testing. Additionally, this decreased overall patient wait times and reduced delays from the first antenatal visit to ART initiation, which HCWs described as better for their patients:

“There are less processes or activities done before a patient is initiated. In other words a patient is easily initiated into ART and other investigations are done later thus not putting the patient at risk while waiting for lab results.” (Nurse midwife)

“Initiating without CD4+ and lab results has been easier because previously we would have a challenge of CD4+ machine not functioning well & transport issues to fetch results, delaying initiation.” (Peer counselor)

“It has cut down the movement of clients in that they have to return to facility a lot to collect labs before initiation” (Nurse midwife)

“Same day initiations help avoid losing clients who may otherwise be lost.” (Nurse midwife)

#### Easy to explain to patients

Additionally, the advantages of immediate ART initiation were felt within the patient-provider interaction, as HCWs described Option B+ as easy to explain to patients, given that the protocol was simply to start all HIV-positive mothers on ART immediately. HCWs reported that because a lifelong ART regimen for all HIV-positive pregnant mothers was simpler for patients to understand, initiating women on PMTCT under Option B+ was also easier for HCWs:

“It’s easier to teach women on taking medication there are no options for the women to choose from. It saves time.” (Mother mentor)

“Our goal as health care workers is for the mothers to give birth to an HIV negative baby and that the mother remain healthy. So it’s easy because moms are initiated as soon as they test positive and unlike AZT they take them life long, hence shorter and simpler messages.” (Mother mentor)

“The packaging is easier for the clients & less complicating now in terms of the meds they get.” (Senior nurse)

Healthcare workers also indicated that patient awareness had increased since the national rollout of Option B+, which occurred between the two month and two year interviews, simplifying their interactions with patients. This was noted to potentially be the result of all clinics implementing the same option:

“All health facilities now offer Option B+. We are now all speaking one language.” (Peer counselor)

“All pregnant women are now aware that when positive you initiate lifelong ART, we believe transmission is decreasing.” (Nurse midwife)

“We talk of one thing now to pregnant positive women that ART is the way to go.” (Mentor mother)

#### Simple and effective counseling

Counseling under Option B+ was described by HCWs as simple and effective, due in part to the straightforwardness of one course of treatment, ART for life. Some HCWs attributed the effectiveness of the counseling to the comprehensiveness of the counseling protocol, as well as the inclusion of flip charts for Option B+ education.

“Counseling becomes easier since there are no more options to choose from.” (Nurse midwife)

“The teaching includes everything. Then when counseling after the morning class becomes easier because they have learnt.” (Mother mentor)

“Option B+ has reduced time spent on counseling. We just enroll. It has reduced our workload. You know you will just be talking about pills.” (Mentor mother)

“A majority of women have agreed to start ART same day. This is due to the quality counseling that has been strengthened by the availability of cue cards.” (Peer counselor)

#### Decreased institutional barriers to care

HCWs were asked about specific barriers to PMTCT uptake at both two months and two years post-transition. Several HCWs mentioned improved coordination between services as an advantage of Option B+:

“Comprehensive services are provided under Option B+, women get everything under one roof and we health care workers do not move around. Healthcare workers work with one client and provide them with services.” (Peer counselor)

“Patients get all the drugs (ANC and ART) in the same room and nurses do not have to use prescription forms that patients would have to take to the pharmacy.” (Nurse midwife)

Additionally, the number of HCWs who cited lack of coordination between services as a barrier to PMTCT uptake decreased significantly over two years (Table 3). Direct comparisons between HCWs who completed surveys at both two months and two years post-transition (*n* = 35) show that only 6% of HCWs cited lack of coordination between services (requiring women to visit multiple service points) as a barrier to PMTCT uptake two years after transition, compared to 46% of HCWs at two months post-transition.

**Table 3:** Differences in perceived institutional barriers to PMTCT care at two months and two years post-transition to Option B+^a^

	2 months post-transition (*N* = 35)	2 years post-transition (*N* = 35)	*p*-value
*Yes/No, Yes reported*	*N*(%)	*N*(%)	
Clinic runs out of supplies and medications that women need	8 (23%)	1 (3%)	0.0391
Too many other people at the clinic	23 (66%)	12 (34%)	0.0074
Facility is too far	16 (46%)	6 (17%)	0.0309
Appointment system does not work	9 (26%)	0 (0)	n/a
Waiting time is too long	27 (77%)	14 (40%)	0.0002
Attitudes of health care workers	25 (71%)	8 (23%)	< 0.0001
No evening or weekend hours	24 (69%)	15 (43%)	0.0225
No coordination between services (services not integrated so woman has to visit too many service areas/clinics to receive care)	16 (46%)	2 (6%)	0.0013
Clinic staff don’t spend enough time with patient	11 (31%)	2 (6%)	0.0039
Clinic staff have too many other things to do	25 (71%)	6 (17%)	< 0.0001
Clinic staff don’t provide sufficient counseling support	9 (26%)	1 (3%)	0.0078

^a^Includes only healthcare workers who participated in both surveys at two-months and two-years post-transition

Finally, direct comparisons also demonstrate that compared to two months post-transition, fewer HCWs reported institutional barriers to care after two years, including: inadequate appointment systems (26% vs. 0%), lack of supplies (23% vs. 3%), overcrowded clinics (66% vs. 34%), long wait times (77% vs. 40%), attitudes of healthcare workers (71% vs. 23%), overworked staff (71% vs. 17%), and lack of counseling support (26% vs. 3%).

### Challenges to Option B+

#### Increased worker burden

Although fewer HCWs reported worker burden as an institutional barrier to PMTCT care after two years, a majority of HCWs reported that their workloads increased under Option B+, and this remained consistent over time. Sixty-two percent of HCWs at two months post-transition and 68% of HCWs at two years post-transition reported more work under Option B+ compared to Option A (Table 2), specifically noting increased involvement in adherence counseling, prescribing/monitoring medications, and appointment scheduling/tracking since the transition to Option B+. Some HCWs attributed their higher workloads to increased client loads, now that all HIV-positive women were initiated on ART.

“A majority of clients are now enrolling into lifelong ART, [leading to] more workload for initiations.” (Nurse midwife)

“We enroll more women regardless of CD4+ count, [there is] more client load. Before we waited for eligibility based on CD4+ count.” (Nurse midwife)

Additionally, HCWs noted the substantial volume of required paperwork under Option B+ as a contributing factor to their increased workloads. While 56% of HCWs reported that documentation under Option B+ was easy, 42% of HCWs found documentation under Option B+ to be difficult (Table 2), and of those, nearly all said that there was too much paperwork. HCWs described a vast amount of required forms and registers, often front-loaded at the initiation appointment, which could create mistakes/gaps in documentation:

“Too much paper, too many registers, CCF, prescription form, ANC register, ANC card, Pre-ART register, & ART register.” (Nurse midwife)

“We spend too much time initiating one woman. Opening a file, counseling. It’s more work, you have to appoint two weeks review. There is too much paperwork.” (Mentor mother/Site coordinator)

“You spend longer time with the client, counseling them to initiate, yet we have a lot of clients that need our attention. This also leads to poor documentation.” (Nurse midwife)

#### New patient-level barriers to ART uptake

The most frequently cited challenge to Option B+ at both two months and two years post-transition was that same-day ART initiation introduced new, more immediate patient-level barriers to the acceptability of ART uptake. The majority of HCWs at two months and two years post-transition reported that the acceleration of ART initiation presented barriers to acceptability for patients who are hesitant to start treatment immediately. Patients may require time to consult with or disclose their status to their partners and/or family members before starting ART, and same-day initiation does not allow patient this time. Additionally, patients might not be ready to disclose their status to their partners and/or family members.

“They feel unready, need more time to think about it because it’s a lifetime commitment and need to consult husbands/partners.” (Nurse midwife)

“Disclosure usually becomes a problem as clients have to go and disclose to family members.” (Expert client)

“They fear taking ARVs home before consulting with their partners. Others are in denial, refuse that they take pills for life.” (Mentor mother)

“They want to go and think about it… They do not want to take ART for life. They have not disclosed to partners.” (Mentor mother)

Healthcare workers noted that some patients might feel unready to begin lifelong drug therapy, particularly if they have been diagnosed as HIV-positive that same day. The immediacy of same-day initiation may overwhelm women who have multiple factors to consider and accept before being able to commit to lifelong treatment:

“As you counsel the woman on the day they test positive and that they have to start ART, it is hard to tell the woman to accept the status and take pills that same time too.” (Mentor mother)

“It’s not easy to counsel clients who have just been diagnosed HIV-positive, there is no room for her to digest and accept status.” (Nurse midwife)

“It is all too overwhelming for some clients. Testing today, taking ART today, having to disclose to partner.” (Nurse)

Some HCWs explained that patients who are already familiar with Option A might not understand the need to commit to lifelong treatment:

“Because now we are talking about lifelong treatment when they aren’t sick yet. They have been used to short-term AZT. Changing that mentality is difficult.” (Senior nurse)

Even in light of new patient-level barriers to Option B+, after two years fewer HCWs (14%) reported that women commonly refuse to initiate ART on the same day as diagnosis, compared to two months (32%) (Table 2).

#### Retention, adherence, and follow-up

Two years after transitioning to Option B+, HCWs noted that retention and adherence under Option B+ was difficult, explaining that patients may agree to initiate ART during the clinic visit to please the HCWs, but later fail to adhere to their medication or return to the clinic. Women also may take the medication while they are pregnant, but once the baby is born they fail to return for care.

“We give the woman the package but you never know if they really take it, some just don’t come back.” (Mentor mother)

“We are not always sure that the clients take the medication or they initiate to please us.” (Nurse midwife)

HCWs reported that it was hard to trace women who have missed their scheduled appointments. Forty-two percent of HCWs reported that following up with patients was difficult at two years post-transition (Table 2). Healthcare workers described patients who would leave with the medication but never return to clinic, and some noted that patients might not return because they were not ready to begin lifelong ART:

“Some will only take the medication in front of you then leave for good.” (Nurse)

“Its not easy to follow them up because we initiate them first day when they hardly know us and they find it hard to talk about all their issues. They feel like we have forced them into this.” (Expert client)

“They just don’t come. I think maybe the start is sudden.” (Nurse midwife)

Other HCWs attributed difficult follow-up to high patient mobility, noting that patients sometimes relocated without notifying the clinic of their new contact details.

“The clients are very mobile, they change houses frequently or lose their job leaving us with no forwarding address.” (Nurse midwife)

“Most of the clients are very mobile due to the fact that we in an industrial area the clients are here for work mainly, if contract ends they relocate.” (Nurse midwife)

“[Some patients] travel a lot so they don’t come and we can’t confirm if they are continuing with services there [at their new location].” (Mentor mother)

Patient mobility was also framed as a significant barrier to retention and adherence in PMTCT services after two years. HCWs reported that changes in women’s living situations after delivery made it difficult for women to continue their care.

“They drop out due to lack of finances and change of residential area, also after delivery she is surrounded by people she has not disclosed to and then find it hard to adhere to her treatment or baby’s treatment. Some babies are separated from the mother at delivery leading to poor adherence; mom feels there’s no more reason to come because her goal was bringing the baby.” (Nurse midwife)

“After delivery women move back home to the rural areas. They change health facilities.” (Expert client)

“[Some patients] move to different areas and are afraid to be known as HIV positive.” (Nurse)

“[In] most cases, they came here for work so after delivery they take babies to their residential homes to stay with the grandmother.” (Mentor mother)

## Discussion

This study demonstrates that healthcare workers in this setting view Option B+ as an acceptable and feasible PMTCT approach and identifies advantages and challenges of Option B+ from the perspectives of healthcare workers. Our study contributes to the growing body of literature on HCW experiences [13, 24, 26–32] and provides critical insight to the experiences of HCWs over time with the Option B+ approach.

Earlier findings from data collected at the first time point (prior to transition to Option B+) suggested that HCWs viewed Option B+ as an acceptable and feasible PMTCT approach, highlighting improved coordination between PMTCT services, stronger counseling and follow-up procedures and reduced wait time for ART initiation. However, many also cited possible concerns related to Option B+ implementation. HCWs mentioned issues related to client acceptability including lack of readiness to start ART, prior familiarity with Option A and challenges around disclosure and consultation with family as possible barriers to implementation of Option B+. In addition, increased worker burden with more women initiating ART and requiring management on treatment was cited as a concern [24].

Study findings demonstrate that by eliminating ART eligibility requirements for HIV-positive pregnant women, Option B+ offers important benefits to both HCWs and patients, including reduced wait times, improved service coordination and delivery, decreased institutional barriers to care, and increased ease with patients. Study findings also reveal that as Option B+ increases the number of patients eligible for PMTCT, HCWs face increased workloads and manage more job responsibilities. While immediate ART initiation provided many benefits for HCWs and patients, it also produced new patient-level barriers to care, such as being unready to begin lifelong ART the same day as receiving an HIV diagnosis, and intensified established patient-level barriers to care, such as partner approval and/or disclosure. Study findings highlight the important challenges in retention and adherence under Option B+, especially for HCWs who are already burdened with increased patients and workloads. While Option B+ is successful in simplifying PMTCT services for HCWs and patients, this study highlights the range of challenges faced by HCWs and demonstrates the practical issues stemming from inadequacies in the health system infrastructure, including overburdened HCWs as a result of HCW shortages. These findings are particularly relevant as countries expand Test and Start approaches beyond PMTCT to include all individuals living with HIV.

### Advantages of Option B+

Nearly all HCWs discussed the elimination of laboratory testing associated with Option A as an important advantage of Option B+ at both two months and two years post-transition. The benefits of reduced testing resonated at multiple levels of the healthcare facilities, and many HCWs noted that the elimination of CD4+ testing for ART initiation helped to alleviate the service delivery traffic jams that occurred under Option A, namely limited resources and high demand for CD4+ testing. Without the need for lab tests, HCWs reported that Option B+ was easier to coordinate and wait times were reduced. Rapid ART initiation is particularly important during pregnancy as most transmission occur late in gestation and during labor and viral load is the critical determinant of transmission risk [33]. Under Option B+, women were free to initiate ART immediately, thus reducing the risk for mother-to-child transmission [34] as well reducing the number of steps in the pretreatment cascade and opportunities for disengagement from care. HCWs also noted that the Option B+ regimen was easy to explain and easier for patients to understand. After two years, HCWs reported decreased institutional barriers to care under Option B+, such as lack of supplies or long wait times. These findings indicate that Option B+ may somewhat alleviate previous facility-level barriers to care such as lack of quick CD4+ testing, long wait times, complicated treatment protocols, communication and/or coordination problems, and other health systems-related constraints [14, 18, 35]. Additionally, the high acceptability of Option B+ among HCWs and the resulting increased ease with patients due to the streamlined process of Option B+ may have the potential to improve patient-provider interactions, which are valued by patients and have been shown to affect the likelihood of ART initiation, retention, and adherence [18]. Finally, prior research has established that coordination problems in service delivery can delay and/or disrupt patient engagement in the PMTCT cascade [35]. This study found that fewer HCWs reported lack of coordination between services as a barrier to PMTCT uptake over time, indicating that Option B+ may increase the integration of services.

### Challenges to Option B+

Study findings also revealed challenges to Option B+. The majority of HCWs reported increased workloads under Option B+, which was consistent over time. Of those that reported increased work, nearly all cited a vast amount of paperwork and documentation. Previous research has identified paperwork as an ongoing challenge to PMTCT programs [32, 36–38], noting that the multiple registers in PMTCT programs may constitute “a large volume of paperwork, with duplicated information, and a consequent burden on staff workload” [37]. HCWs in this study felt that documentation under Option B+ was often frontloaded, leading to a large amount of paperwork during the first appointment, and some HCWs noted this could potentially lead to inconsistent or inaccurate documentation. HCWs also attributed more work to the increased numbers of clients initiating ART and retained in care under Option B+; this is confirmed in the findings of the parent study, which found the number of women initiating ART at the first antenatal visit increased from 18 (1%) to 902 (86%) at participating study sites [23]. Study findings also showed increasing HCW involvement in a range of job responsibilities within the clinic and indicated that services may have become more integrated over time. Increased coordination between services has been associated with increased workloads for healthcare workers [35, 38, 39]. Additionally, the benefits of increased coordination between services or integrated care can be diminished by other health systems challenges such as human resource shortages, inadequate infrastructure, or poor training [35, 38].

Patient-level barriers to PMTCT uptake were a key concern among most HCWs. Without time for patients to consult with or disclose to their families, HCWs expressed concern that patients may feel overwhelmed by the immediacy of Option B+ Test and Start approach. These findings are supported by recent research on Option B+ in eSwatini which shows women often felt reluctant to start lifelong ART so soon, and when feeling healthy [40]. Although at two years, few HCWs (14%) in this study reported that it was common for women to refuse to initiate ART on the same day as being diagnosed with HIV, most HCWs reported patient-level barriers such as partner disclosure and consulting as a point of difficulty with same-day ART initiation under Option B+. A number of previous studies have established the importance of partner disclosure and involvement in PMTCT uptake [18, 41], and these findings contribute to understanding the temporal urgency of these issues under the Option B+ approach. Additionally, patient-level barriers related to partner disclosure and involvement may resonate throughout the PMTCT treatment cascade. A 2014 study in Malawi found that patients who initiated ART on the same day as their HIV diagnosis had the highest rates of loss to follow-up, which researchers attributed to patients being unready to begin lifelong drug therapy, as well as issues with partner disclosure [4]. Together these findings provide important insights to inform country programs as they scale up universal ART employing the Test and Start approach [20].

Finally, study findings indicate that HCWs recognize the specific challenge to adherence and retention among women initiating lifelong ART. While major progress has been made in PMTCT scale-up, Option B+ requires continued engagement and retention in lifelong care, and current studies suggest that 25 to 50% of women may be lost from care at some point during the postpartum period [4, 42, 43]. There is an urgent need to better understand the factors involved in retaining women in lifelong HIV care [42–46]. Several HCWs in this study mentioned high patient mobility as a challenge to retention and care; patient mobility has previously been linked with high rates of attrition in care. It has been established that pregnant women often return to their rural homes after delivery to receive care from their family, potentially leading to a host of associated issues with disclosure, medication adherence, and retention in care [3].

### Limitations

Several study limitations should be noted. As discussed earlier, although we made every attempt to include the same HCW sample in both post-transition survey time points, the two-year post-transition sample included 15 replacement participants, generally due to staff turnover. However, the general distribution of HCW job responsibilities across the two-month and two-year post-transition time points was largely unchanged. It should also be noted that in 2014, eSwatini endorsed Option B+ for PMTCT, at which point all health facilities in the country transitioned to the Option B+ approach. Therefore, when HCWs were surveyed at the two-year post-transition time point, Option B+ was already in place as the standard of care in health facilities across eSwatini. It is likely that the national rollout of Option B+ affected the experiences of HCWs at study facilities. Healthcare workers in this study were also provided with support from study staff before transition and during the study. Thus, HCW experiences with Option B+ within this study may not be representative of HCW experiences with Option B+ at other facilities within eSwatini.

This study also has several strengths. Few studies have explored the acceptability of Option B+ among HCWs; this study provides important insight on Option B+ acceptability among this group. By including both close-ended and open-ended survey questions, this study was able to measure HCW acceptability of Option B+ while also capturing the nuance and depth of HCW experiences with Option B+. Finally, to our knowledge, this is the first study to evaluate HCW experiences with an intervention over a two-year study duration.

## Conclusions

Option B+ offers important advantages to PMTCT service delivery for both healthcare workers and patients. In many ways, Option B+ alleviates worker- and facility-level burdens associated with Option A and streamlines PMTCT services for patients and workers. However, Option B+ also presents its own unique set of challenges. At the patient level, new barriers to ART have emerged or intensified with the immediacy of ART initiation under Option B+. At the facility level, Option B+ has increased the burden on HCWs as their responsibilities expand to adjust for the increase in client enrollment and transformation of PMTCT services from a time-limited intervention into an ongoing practice of care, with specific challenges related to retention, adherence, and follow-up.

Further strengthening of the healthcare system and possible streamlining or consolidation of documentation will be necessary to alleviate worker burden and ensure effective support and monitoring of client adherence and retention. Moving forward, HCW perceptions and experiences with Option B+ should inform ongoing PMTCT scale-up and be considered more broadly as countries implement Option B+ and move towards universal treatment for all HIV-positive individuals.

## Additional files

Additional file 1: HCW Acceptability Follow-up Questionnaire, 10 Sept 2013. Questionnaire administered to HCWs at 2-months post Option B+ transition. (PDF 361 kb)

Additional file 2: HCW Acceptability Second Follow-up Questionnaire, 26 May 2015. Questionnaire administered to HCWs at 2-years post Option B+ transition. (PDF 320 kb)

## Abbreviations

APS: Adherence/psychosocial
ART: Antiretroviral therapy
ARV: Antiretroviral medication
AZT: Zidovudine
HCW: Health Care Worker
MNCH: Mother, newborn and child health
PMTCT: Prevention of mother-to-child transmission
WHO: World Health Organization
