# Supplementary material for: Healthcare worker experiences with Option B+ for prevention of mother-to-child HIV transmission in eSwatini: findings from a two-year follow-up study
Source: BMC Health Serv Res. 2019 Apr 2;19:210. doi: 10.1186/s12913-019-3997-1 (PMC6444445; doi:10.1186/s12913-019-3997-1)
Supplement: Supplementary file 1 — HCW Acceptability Follow-up Questionnaire, 10 Sept 2013. Questionnaire administered to HCWs at 2-months post Option B+ transition. (PDF 361 kb) [file 12913_2019_3997_MOESM1_ESM.pdf]

SITUKULWANE LESIPHEPHILE - SAFE GENERATIONS

PMTCT Health Care Worker Acceptability Assessment

COMPLETE ALL THE INFORMATION ON THIS PAGE BEFORE STARTING THE INTERVIEW.

Interviewer code	☐☐☐
HCW participant identification number	☐☐-☐☐☐☐
Date of interview (Study enrollment date)	☐☐ ☐☐ ☐☐☐☐<br>DAY MONTH YEAR
Timing of Follow-up Interview	_____ months post implementation of B+
Study Site	☐ KSII PHU<br>☐ RFM Hospital<br>☐ FLAS Manzini<br>☐ Siteki PHU<br>☐ MSF Matsapha<br>☐ Mbikwakhe<br>☐ Siphofaneni<br>☐ Lamvelase<br>☐ Luyengo<br>☐ Mankayane PHU

Columbia University IRB
IRB-AAAL0661
IRB Approval Date: 01/22/2016
for use until: 01/21/2017

Introduction:
Currently, HIV-positive pregnant women who come to this clinic for PMTCT services receive medication to prevent their babies from getting the HIV virus. Some women get AZT and others get lifetime treatment (ART) depending on their CD4 count (This is called Option A.) While many women do receive some PMTCT services, there are a lot of challenges and many women aren't able to come back to the clinic or can't take all of the necessary steps to keep themselves and their baby healthy during their pregnancy, and after they deliver. With these problems in mind, the Ministry of Health of the Kingdom Swaziland has decided to pilot another PMTCT approach where all HIV-positive pregnant women start ART and stay on treatment for life whether they have a low or high CD4 count (This is called Option B+). Starting next month all women coming for PMTCT services at this clinic will receive this Option B+.

We are planning to evaluate how this new PMTCT approach, Option B+, is working. As part of the evaluation, we are going to be asking health care workers, like you, about their experience providing PMTCT services at this clinic. We would like to hear about your experience with Option A and later, we will ask you about your experience with Option B+ after it has been implemented at this clinic. In addition, we will ask you some questions about yourself, HIV in general and PMTCT.

Section I. DEMOGRAPHICS

Basic Information

1. How old are you?	Age: ___________(years)
2. Gender?	☐ Female<br>☐ Male
3. What is your area of residence?	*Area of residence:*<br>____
4. What is your current job?	☐ Nurse<br>☐ Nurse Midwife<br>☐ Expert Client<br>☐ Peer Counselor<br>☐ Other____________________
5. Where do you work? *(circle all that apply)*	*Specify:*<br>☐ Antenatal Care services (ANC)<br>☐ Maternal Child Health services (MCH)<br>☐ Hospital Ward (Adult)<br>☐ Hospital Ward (Pediatric)<br>☐ Other:____________________<br>☐ Child welfare services<br>☐ Pediatric ART Clinic<br>☐ Adult ART Clinic

Columbia University IRB
IRB-AAAL0661
IRB Approval Date: 01/22/2016
for use until: 01/21/2017

6. In your role as [**INSERT RESPONSE from Question 4**], what types of activities are you involved with?<br><br>*(check all that apply)*	☐ HIV testing and giving HIV results<br>☐ CD4 testing and giving CD4 results<br>☐ Counseling on PMTCT<br>☐ Counseling on infant feeding<br>☐ Counseling on antenatal care<br>☐ Counseling on adherence<br>☐ Prescribing HIV medications<br>☐ Monitoring side effects from medications<br>☐ PCR testing<br>☐ Counseling on family planning<br>☐ Scheduling appointments<br>☐ Following-up missed appointments<br>☐ Other, specify: __________________________

Columbia University IRB
IRB-AAAL0661
IRB Approval Date: 01/22/2016
for use until: 01/21/2017

**Section II. UNDERSTANDING OF OPTION B+**				
7. How long have you been involved in the implementation of Option B+ at this clinic?	*Length of time:* _____________months			
8. *I am going to read a list and would like you to tell me which items on the list are part of Option B+ for PMTCT. Please say TRUE if part of Option B+ and FALSE if not part of Option B+ for PMTCT.*				
Sending a CD4 count test	True	False	Refuse	Don’t Know
Making an appointment for women in the ART clinic	True	False	Refuse	Don’t Know
Giving ART to women with CD4<350	True	False	Refuse	Don’t Know
Giving AZT to women with CD4>350	True	False	Refuse	Don’t Know
Starting AZT/ART 14 weeks gestation	True	False	Refuse	Don’t Know
Giving ART to all women	True	False	Refuse	Don’t Know
Starting ART as soon as diagnosed as HIV infected	True	False	Refuse	Don’t Know
Starting ART for life for all HIV positive pregnant women	True	False	Refuse	Don’t Know
Giving NVP to women to take at delivery	True	False	Refuse	Don’t Know
Giving NVP to babies after birth	True	False	Refuse	Don’t Know
Testing infants with DNA PCR DBS	True	False	Refuse	Don’t Know
Counseling mothers on breast feeding	True	False	Refuse	Don’t Know
Testing partners	True	False	Refuse	Don’t Know
Testing other family members	True	False	Refuse	Don’t Know
Checking CD4 counts after delivery	True	False	Refuse	Don’t Know
Other___________________________				

Columbia University IRB
IRB-AAAL0661
IRB Approval Date: 01/22/2016
for use until: 01/21/2017

9. In your opinion what are the things about Option B+ that are **easier** for you and your co-workers to implement compared to Option A?	______<br>______<br>______<br>______<br>______
10. In your opinion what are the things about Option B+ that are **more difficult** for you and your co-workers to implement compared to Option A?	______<br>______<br>______<br>______<br>______
11. In your opinion is it easier or more difficult to counsel patients in Option B+ compared to Option A?	☐ Easier<br>☐ More difficult *(specify why)*______<br>______
12. What do you discuss when you counsel HIV-positive women about ART for life?	______<br>______<br>______<br>______<br>______
13. Do you think you have more work or less work now (since implementation of Option B+)?	☐ More work<br>☐ Less work<br>☐ Same amount<br>☐ Don't know<br>☐ Other:______<br>______

Columbia University IRB
IRB-AAAL0661
IRB Approval Date: 01/22/2016
for use until: 01/21/2017

14. We know that sometimes HIV positive women don't complete all of the PMTCT steps in pregnancy and after the baby is born. What do you think are some of the barriers HIV positive women now face with the implementation of Option B+?	______
15. Do you find that women commonly refuse to start ART the same day they are diagnosed with HIV?	☐ No<br>☐ Yes *(specify why)* ______

Columbia University IRB
IRB-AAAL0661
IRB Approval Date: 01/22/2016
for use until: 01/21/2017

Section III. ATTITUDES ABOUT PMTCT AND PERCEPTIONS OF CLIENT UPTAKE

I will read a few statements regarding attitudes and perceptions of PMTCT Clients. Please respond with Agree with statement, Disagree with statement or Neutral

Read Statements	*Circle one response*				
16. It is very difficult for an HIV positive woman to decide to start taking HIV medicines for life while she is pregnant.	Agree	Disagree	Neutral	Don’t Know	Refused
17. It is very difficult for an HIV positive pregnant woman to decide to start taking HIV medicines for life if she is feeling healthy.	Agree	Disagree	Neutral	Don’t Know	Refused
18. It is very difficult for an HIV positive pregnant woman with a high CD4 count to decide to start taking HIV medicines for life.	Agree	Disagree	Neutral	Don’t Know	Refused
19. HIV-positive pregnant women should start HIV medicine (ART) and remain on it for life rather than giving ART to only those who have a low CD4 count and giving ARV prophylaxis to those with high CD4.	Agree	Disagree	Neutral	Don’t Know	Refused
20. It will be difficult to explain why a healthy HIV positive pregnant woman should start HIV medicines when other adults don’t start until their CD4 count is low.	Agree	Disagree	Neutral	Don’t Know	Refused
21. It is easier to counsel patients under option A compared to option B+.	Agree	Disagree	Neutral	Don’t Know	Refused

Columbia University IRB
IRB-AAAL0661
IRB Approval Date: 01/22/2016
for use until: 01/21/2017

**Section IV. BARRIERS TO UPTAKE OF PMTCT**	
Sometimes women stop coming back to the clinic for PMTCT services. We want to learn more about why this happens. Could any of these be a reason why a woman would not come back for PMTCT services?	
22. Problems with the health services<br><br>*(read responses and tick all that apply)*	☐ Clinic runs out of supplies and medications that women need<br>☐ Too many other people at the clinic<br>☐ Facility is too far<br>☐ Appointment system does not work<br>☐ Waiting time is too long<br>☐ Attitudes of health care workers<br>☐ No evening or weekend hours<br>☐ No coordination between services (services not integrated so woman has to visit too many service areas/clinics to receive care)<br>☐ Other, specify:______________________________
23. Problems with the clinic staff<br><br>*(read responses and tick all that apply)*	☐ Clinic staff don't spend enough time with patient<br>☐ Clinic staff don't seem well trained<br>☐ Clinic staff have too many other things to do<br>☐ Clinic staff don't provide sufficient counseling support<br>☐ Other, specify ______________________________

Columbia University IRB
IRB-AAAL0661
IRB Approval Date: 01/22/2016
for use until: 01/21/2017

24. Personal issues for women<br><br>(*read responses and tick all that apply)*	☐ Concerned about confidentiality<br>☐ Concerned about others learning their HIV status<br>☐ Don't want to start taking HIV medicines during pregnancy<br>☐ Don't want to start taking HIV medicines when they are feeling healthy<br>☐ Don't want to take HIV medicines during breastfeeding<br>☐ Don't believe they have HIV infection<br>☐ Afraid of side effects from taking HIV medicines<br>☐ Pressure from family members including husband/partner *(specify pressure)*____<br>____<br>☐ Other, specify: ____

Columbia University IRB
IRB-AAAL0661
IRB Approval Date: 01/22/2016
for use until: 01/21/2017

**Section V. INFANT FEEDING**					
Beliefs *I will read a few statements regarding infant feeding in the context of HIV. Please respond with Agree with statement, Disagree with statement or Neutral*					
Read Responses	*Circle one response*				
25. It is safe for HIV-positive women to breastfeed their infant.	Agree	Neutral	Disagree	Don't Know	Refused
26. Formula is the preferred way for an HIV-positive woman to feed her baby.	Agree	Neutral	Disagree	Don't Know	Refused
27. It is safe for HIV-positive mothers who are taking HIV medicines to breastfeed their infants.	Agree	Neutral	Disagree	Don't Know	Refused
28. It safe to give HIV medicines to babies who are born to HIV-positive mothers.	Agree	Neutral	Disagree	Don't Know	Refused
29. Babies who are younger than 6 months of age should be given nothing else to drink or eat aside from breast milk.	Agree	Neutral	Disagree	Don't Know	Refused

Columbia University IRB
IRB-AAAL0661
IRB Approval Date: 01/22/2016
for use until: 01/21/2017

Infant Feeding Practices					
30. Counseling HIV-positive women about how to feed their infants is the same whether the women is receiving Option A or B+.	Agree	Neutral	Disagree	Don’t Know	Refused
31. When should an HIV-positive mother stop breastfeeding?		*Age of child:*<br>Months_______________			
32. What do you discuss when you counsel HIV-positive women about feeding their infants?<br><br>*(Do not read responses aloud. Check all that apply)*		☐ ARVs during breastfeeding<br>☐ Exclusive breastfeeding<br>☐ Introduction of complementary foods<br>☐ Breast health<br>☐ Frequency of feeds<br>☐ Latch<br>☐ Breastfeeding problems<br>☐ Poor feeding<br>☐ Growth monitoring<br>☐Other:________________________________			

Columbia University IRB
IRB-AAAL0661
IRB Approval Date: 01/22/2016
for use until: 01/21/2017

**Section VI. ADDITIONAL INFORMATION**
33. Is there anything else that you would like to share about your experience with PMTCT services? Are there ways that these services could be improved?

Columbia University IRB
IRB-AAAL0661
IRB Approval Date: 01/22/2016
for use until: 01/21/2017
