# Supplementary material for: Healthcare worker experiences with Option B+ for prevention of mother-to-child HIV transmission in eSwatini: findings from a two-year follow-up study
Source: BMC Health Serv Res. 2019 Apr 2;19:210. doi: 10.1186/s12913-019-3997-1 (PMC6444445; doi:10.1186/s12913-019-3997-1)
Supplement: Supplementary file 2 — HCW Acceptability Second Follow-up Questionnaire, 26 May 2015. Questionnaire administered to HCWs at 2-years post Option B+ transition. (PDF 320 kb) [file 12913_2019_3997_MOESM2_ESM.pdf]

SITUKULWANE LESIPHEPHILE - SAFE GENERATIONS

PMTCT Health Care Worker Acceptability Assessment

COMPLETE ALL THE INFORMATION ON THIS PAGE BEFORE STARTING THE INTERVIEW.

Interviewer code	☐☐☐
HCW participant identification number	☐☐-☐☐☐☐
Date of interview (Study enrollment date)	☐☐ ☐☐ ☐☐☐☐<br>DAY MONTH YEAR
Timing of Follow-up Interview	_____ months post implementation of B+
Study Site	☐ KSII PHU<br>☐ RFM Hospital<br>☐ FLAS Manzini<br>☐ Siteki PHU<br>☐ MSF Matsapha<br>☐ Mbikwakhe<br>☐ Siphofaneni<br>☐ Lamvelase<br>☐ Luyengo<br>☐ Mankayane PHU

Columbia University IRB
IRB-AAAL0661
IRB Approval Date: 01/22/2016
for use until: 01/21/2017

Introduction:

Recently, the Ministry of Health of the Kingdom Swaziland has implemented a PMTCT approach where all HIV-positive pregnant women start ART and stay on treatment for life whether they have a low or high CD4 count (Option B+). Several months ago, as part of a research study, all women coming for PMTCT services at this clinic started receiving Option B+ instead of Option A.

We want to evaluate how this new PMTCT approach, Option B+, is working. As part of the evaluation, we are going to be asking health care workers, like you, about their experience providing PMTCT services at this clinic.

Section I. DEMOGRAPHICS

Basic Information

1. How old are you?	Age: ___________(years)
2. Gender?	☐ Female<br>☐ Male
3. What is your area of residence?	*Area of residence:*<br>____
4. What is your current job?	☐ Nurse<br>☐ Nurse Midwife<br>☐ Expert Client<br>☐ Peer Counselor<br>☐ Other____________________
5. Where do you work?<br>*(check all that apply)*	*Specify:*<br>☐ Antenatal Care services (ANC)<br>☐ Maternal Child Health services (MCH)<br>☐ Hospital Ward (Adult)<br>☐ Hospital Ward (Pediatric)<br>☐ Other:____________________<br>☐ Child welfare services<br>☐ Pediatric ART Clinic<br>☐ Adult ART Clinic

Columbia University IRB
IRB-AAAL0661
IRB Approval Date: 01/22/2016
for use until: 01/21/2017

6. In your role as [**INSERT RESPONSE from Question 4**], what types of activities are you involved with?<br><br>*(check all that apply)*	☐ HIV testing and giving HIV results<br>☐ CD4 testing and giving CD4 results<br>☐ Counseling on PMTCT<br>☐ Counseling on infant feeding<br>☐ Counseling on antenatal care<br>☐ Counseling on adherence<br>☐ Prescribing HIV medications<br>☐ Monitoring side effects from medications<br>☐ PCR testing<br>☐ Counseling on family planning<br>☐ Scheduling appointments<br>☐ Following-up missed appointments<br>☐ Other, specify: ___________________________

Columbia University IRB
IRB-AAAL0661
IRB Approval Date: 01/22/2016
for use until: 01/21/2017

Section II. UNDERSTANDING OF OPTION B+				
7. When was Option B+ implemented at this clinic?				
8. Were you a part of the transition from Option A to Option B+?				
9. How long have you been involved in the implementation of Option B+ at this clinic?	*Length of time:* ______________months			
10. *I am going to read a list and would like you to tell me which items on the list are part of Option B+ for PMTCT. Please say TRUE if part of Option B+ and FALSE if not part of Option B+ for PMTCT.*				
Sending a CD4 count test	True	False	Refuse	Don't Know
Making an appointment for women in the ART clinic	True	False	Refuse	Don't Know
Giving ART to women with CD4<350	True	False	Refuse	Don't Know
Giving AZT to women with CD4>350	True	False	Refuse	Don't Know
Starting AZT/ART 14 weeks gestation	True	False	Refuse	Don't Know
Giving ART to all women	True	False	Refuse	Don't Know
Starting ART as soon as diagnosed as HIV infected	True	False	Refuse	Don't Know
Starting ART for life for all HIV positive pregnant women	True	False	Refuse	Don't Know
Giving NVP to women to take at delivery	True	False	Refuse	Don't Know
Giving NVP to babies after birth	True	False	Refuse	Don't Know
Testing infants with DNA PCR DBS	True	False	Refuse	Don't Know
Counseling mothers on breast feeding	True	False	Refuse	Don't Know
Testing partners	True	False	Refuse	Don't Know
Testing other family members	True	False	Refuse	Don't Know
Checking CD4 counts after delivery	True	False	Refuse	Don't Know

Columbia University IRB
IRB-AAAL0661
IRB Approval Date: 01/22/2016
for use until: 01/21/2017

11. In your opinion what are the things about Option B+ that are **easy** for you and your co-workers?	
12. In your opinion what are the things about Option B+ that are **difficult** for you and your co-workers?	
13. Do you think following up with patients who initiated ART under Option B+ is easy or hard for healthcare workers?<br><br>Please explain.	☐ Easy *(explain)*<br><br>☐ Hard *(explain)*
14. Do you think documentation of patient care under Option B+ is easy or hard for healthcare workers? Please explain.	Easy *(explain)*

Columbia University IRB
IRB-AAAL0661
IRB Approval Date: 01/22/2016
for use until: 01/21/2017

	☐ Hard *(explain)*
15. <u>Overall</u>, do you think Option B+ is easy or hard for <u>healthcare workers</u>?<br><br>Please explain.	☐ Easy *(explain)*<br><br>☐ Hard *(explain)*
16. Do you think you have more work or less work now (since implementation of Option B+)?	☐ More work<br>☐ Less work<br>☐ Same amount
17. Please explain	

Columbia University IRB
IRB-AAAL0661
IRB Approval Date: 01/22/2016
for use until: 01/21/2017

	______
**COUNSELING**	
18. What do you discuss when you counsel HIV-positive women about ART for life?	______
19. In your opinion is it easy or difficult to counsel patients about Option B+?<br><br>Please explain.	☐ Easy *(explain)* ______<br><br>☐ Difficult *(explain)* ______
**PATIENTS**	

Columbia University IRB
IRB-AAAL0661
IRB Approval Date: 01/22/2016
for use until: 01/21/2017

20. Do you find that women commonly refuse to start ART the same day they are diagnosed with HIV?<br><br>Please explain.	☐ No *(explain)* __________<br>__________<br>__________<br>__________<br>__________<br>☐ Yes *(specify why)* __________<br>__________<br>__________<br>__________<br>__________
21. In your opinion what are the things about Option B+ that are **easy** for patients?<br><br>Please explain.	__________<br>__________<br>__________<br>__________
22. In your opinion what are the things about Option B+ that are **difficult** for patients?<br><br>Please explain.	__________<br>__________<br>__________<br>__________
23. We know that sometimes HIV-positive women do not complete all of the PMTCT steps during pregnancy and after the baby is born. What do you think are some of the common barriers HIV-positive women face in completing these steps under Option B+?	__________<br>__________<br>__________<br>__________<br>__________<br>__________
24. How frequently do women drop out of care before giving	☐ Never

Columbia University IRB
IRB-AAAL0661
IRB Approval Date: 01/22/2016
for use until: 01/21/2017

birth at your facility?	☐ Less than half the time<br>☐ About half the time<br>☐ More than half the time<br>☐ Always
25. Why do you think this happens?	____________________<br>____________________<br>____________________<br>____________________<br>____________________<br>____________________
26. How frequently do women drop out of care <u>after</u> giving birth?	☐ Never<br>☐ Less than half the time<br>☐ About half the time<br>☐ More than half the time<br>☐ Always
27. Why do you think this happens?	____________________<br>____________________<br>____________________<br>____________________<br>____________________
28. How frequently do women attend HIV care visits without their infants (i.e., maternal-infant visits not synchronized)?	☐ Never<br>☐ Less than half the time<br>☐ About half the time<br>☐ More than half the time<br>☐ Always
29. Why do you think this happens?	____________________<br>____________________<br>____________________

Columbia University IRB
IRB-AAAL0661
IRB Approval Date: 01/22/2016
for use until: 01/21/2017

	________________
30. How frequently are babies brought back for care by someone other than their mother?	☐ Never<br>☐ Less than half the time<br>☐ About half the time<br>☐ More than half the time<br>☐ Always
31. Why do you think this happens?	________________<br>________________<br>________________<br>________________<br>________________
32. Overall, do you think Option B+ makes things easy or hard for <u>patients</u>? Please explain.	☐ Easy *(explain)*<br>________________<br>________________<br>________________<br>________________<br>________________<br>☐ Hard *(explain)*<br>________________<br>________________<br>________________<br>________________<br>________________

Columbia University IRB
IRB-AAAL0661
IRB Approval Date: 01/22/2016
for use until: 01/21/2017

Section IV. BARRIERS TO UPTAKE OF PMTCT	
Sometimes women stop coming back to the clinic for PMTCT services. We want to learn more about why this happens.<br>*Could any of these be a reason why a woman would not come back for PMTCT services?*	
33. Problems with the health services<br><br>*(read responses and check all that apply)*	☐ Clinic runs out of supplies and medications that women need<br>☐ Too many other people at the clinic<br>☐ Facility is too far<br>☐ Appointment system does not work<br>☐ Waiting time is too long<br>☐ Attitudes of health care workers<br>☐ No evening or weekend hours<br>☐ No coordination between services (services not integrated so woman has to visit too many service areas/clinics to receive care)<br>☐ Other, specify:____
34. Problems with the clinic staff<br><br>*(read responses and check all that apply)*	☐ Clinic staff don't spend enough time with patient<br>☐ Clinic staff don't seem well trained<br>☐ Clinic staff have too many other things to do<br>☐ Clinic staff don't provide sufficient counseling support<br>☐ Other, specify ____
35. Personal issues for women<br><br>*(read responses and check all that apply)*	☐ Concerned about confidentiality<br>☐ Concerned about others learning their HIV status<br>☐ Want to consult with partner or family<br>☐ Don't want to take HIV medicines for life<br>☐ Don't want to start taking HIV medicines during pregnancy<br>☐ Don't want to start taking HIV medicines when they are feeling healthy<br>☐ Don't want to take HIV medicines during breastfeeding<br>☐ Don't believe they have HIV infection<br>☐ Afraid of side effects from taking HIV medicines<br>☐ Pressure from family members including husband/partner *(specify pressure)*____<br>☐ Other, specify: ______________________________________

Columbia University IRB
IRB-AAAL0661
IRB Approval Date: 01/22/2016
for use until: 01/21/2017

Section III. ATTITUDES ABOUT PMTCT AND PERCEPTIONS OF CLIENT UPTAKE					
I will read a few statements regarding attitudes and perceptions of PMTCT Clients. Please respond with Agree with statement, Disagree with statement or Neutral					
Read Statements	*Circle one response*				
36. It is very difficult for an HIV-positive woman to decide to start taking HIV medicines for life while she is pregnant.	Agree	Disagree	Neutral	Don't Know	Refused
37. It is very difficult for an HIV-positive pregnant woman to decide to start taking HIV medicines for life if she is feeling healthy.	Agree	Disagree	Neutral	Don't Know	Refused
38. It is very difficult for an HIV-positive pregnant woman with a high CD4 count to decide to start taking HIV medicines for life.	Agree	Disagree	Neutral	Don't Know	Refused
39. HIV-positive pregnant women should start HIV medicine (ART) and remain on it for life rather than giving ART to only those who have a low CD4 count and giving ARV prophylaxis to those with high CD4.	Agree	Disagree	Neutral	Don't Know	Refused
40. It will be difficult to explain why a healthy HIV-positive pregnant woman should start HIV medicines when other adults don't start until their CD4 count is low.	Agree	Disagree	Neutral	Don't Know	Refused
41. It is easier to counsel patients under option A compared to option B+.	Agree	Disagree	Neutral	Don't Know	Refused

Columbia University IRB
IRB-AAAL0661
IRB Approval Date: 01/22/2016
for use until: 01/21/2017

Section VI. ADDITIONAL INFORMATION
42. Is there anything else that you would like to share about your experience with PMTCT services? Are there ways that Option B+ services could be improved?

Columbia University IRB
IRB-AAAL0661
IRB Approval Date: 01/22/2016
for use until: 01/21/2017
